# Supplementary material for: Sorting at embryonic boundaries requires high heterotypic interfacial tension
Source: Nat Commun. 2017 Jul 31;8:157. doi: 10.1038/s41467-017-00146-x (PMC5537356; doi:10.1038/s41467-017-00146-x)
Supplement: Supplementary file 2 — Supplementary Software 1 [file 41467_2017_146_MOESM2_ESM.zip › PottsModel/SrcPottsModel/doc/index-files/index-7.html]

G-Index


JavaScript is disabled on your browser.


Skip navigation links


- Overview
- Package
- Class
- Use
- Tree
- Deprecated
- Index
- Help

- Prev Letter
- Next Letter

- Frames
- No Frames

- All Classes

A B C D E F G H I J K L M N O P R S T U V W 


## G

GenerateRandomLattices - Class in simulations


GenerateRandomLattices() - Constructor for class simulations.GenerateRandomLattices


getAggregatedSeries() - Method in class gui.PlotPanel


getAllNeighborPositions() - Method in class model.HexagonPositionManager
:   Returns top, topRight, bottomRight, bottom, bottomLeft, topLeft positions (in this order).

getAllNeighborPositions() - Method in interface model.IPositionManager
:   Defines neighbor positions for given position model.

getAllNeighborPositions() - Method in class model.SquarePositionManager
:   Returns left, bottom, right and top positions (in this order).

getAllNeighbors(Pixel) - Method in class model.HexagonPositionManager


getAllNeighbors(Pixel, Pixel) - Method in class model.HexagonPositionManager


getAllNeighbors(Pixel) - Method in interface model.IPositionManager
:   Returns an array of APixels whose length corresponds to the number of possible positions.

getAllNeighbors(Pixel, Pixel) - Method in interface model.IPositionManager
:   Get union of neighbors of pPixel1 and pPixel2.

getAllNeighbors() - Method in class model.Pixel


getAllNeighbors(Pixel) - Method in class model.SquarePositionManager


getAllNeighbors(Pixel, Pixel) - Method in class model.SquarePositionManager


getAlpha() - Method in class utils.HSLColor
:   Get the Alpha value.

getAngle() - Method in class model.FiniteVector


getAngle() - Method in class model.HalfLine


getAngle() - Method in interface model.Vector


getAngleFromHorizontal(Point, Point) - Static method in class model.VectorUtils
:   Gives angle from horizontal of a point with respect to the

getAngleFromVertex(Point, Point, Point) - Static method in class model.VectorUtils


getArea() - Method in class model.Cell


getAreaEnergy() - Method in class model.Cell
:   Returns area energy such that: energy = areaElasticity \* (area -
    targetArea)^2 If any of targetArea or area are negative, then returns 0.

getAreaEnergy() - Method in class model.CellSet
:   Gets the cumulative area energy of all cells in the system.

getAreaUnit(PixelShape.Type) - Static method in class model.Pixel


getAWTShape() - Method in class gui.Hexagon


getAWTShape() - Method in class gui.PixelDisplay


getAWTShape() - Method in interface gui.PixelShape


getAWTShape() - Method in class gui.Square


getAxis() - Method in class engine.HBLStatistic


getAxis() - Method in class engine.Statistic


getBaseSimulationName() - Method in class model.Constants


getCanvas() - Method in class model.Lattice


getCell(int) - Method in class model.CellSet


getCell() - Method in class model.Pixel


getCellBoundaryEdges() - Method in class model.Pixel
:   Get array of active edges (true) in the same order as getValidEdges()
    defined in PixelShape.

getCellIterator() - Method in class model.Lattice


getCells() - Method in class model.Lattice


getCellType() - Method in class model.Pixel


getCellTypeColor(Cell.CellType) - Static method in class model.Cell


getCellTypeColor() - Method in class model.Cell


getCellTypeColorName(Cell.CellType) - Static method in class model.Cell


getCellTypeFromID(int) - Static method in class model.Cell


getCellTypeInteractionEnergy(Cell.CellType, Cell.CellType) - Method in class model.Constants.ParameterManager


getCenter() - Method in class gui.CellDisplay


getCenter() - Method in class gui.Hexagon


getCenter() - Method in interface gui.PixelShape


getCenter() - Method in class gui.Square


getCenterOfMass() - Method in class model.Cell


getColor() - Method in class model.Pixel


getColorAt(int, int) - Method in class gui.PottsCanvas
:   Get Potts Model Pixel color at position (x, y).

getColumnLabelEnums() - Method in class engine.CellCoordinatesCSVStatistic


getColumnLabelEnums() - Method in class engine.CellShapeCSVStatistic


getColumnLabelEnums() - Method in class engine.CSVStatistic


getColumnLabels() - Method in class engine.CSVStatistic


getComplementary() - Method in class utils.HSLColor
:   Create a RGB Color object that is the complementary color of this
    HSLColor.

getComX() - Method in class model.Cell


getComY() - Method in class model.Cell


getConfigFilename() - Method in class model.Constants


getConnectedNeighborPositions() - Method in class model.HexagonPositionManager


getConnectedNeighborPositions() - Method in interface model.IPositionManager


getConnectedNeighborPositions() - Method in class model.SquarePositionManager


getConnectedNeighbors(Pixel) - Method in class model.HexagonPositionManager


getConnectedNeighbors(Pixel) - Method in interface model.IPositionManager


getConnectedNeighbors() - Method in class model.Pixel


getConnectedNeighbors(Pixel) - Method in class model.SquarePositionManager


getConstants() - Method in class engine.PottsEngine


getConstants() - Method in class engine.Simulation


getConstants() - Method in class model.Cell


getConstants() - Method in interface model.KnowsConstants


getConstants() - Method in class model.Lattice


getConstants() - Method in class model.Pixel


getConstants() - Method in class simulations.BoundarySimulation


getConstants() - Method in class simulations.DefaultSimulation


getConstants() - Method in class simulations.DITHSimulation


getConstants() - Method in class simulations.EndoSimulation


getConstants() - Method in class simulations.FourByFourSimulation


getConstants() - Method in class simulations.GenerateRandomLattices


getConstants() - Method in class simulations.GranerGlazierSimulation


getConstants() - Method in class simulations.NegativeControl


getConstants() - Method in class simulations.SingleCell


getCoordinates() - Method in class gui.Hexagon


getCoordinates() - Method in interface gui.PixelShape


getCoordinates() - Method in class gui.Square


getCoordinates() - Method in class model.Pixel


getCorner(int) - Method in class gui.Hexagon


getCSV(List<XYSeries>) - Method in class gui.PlotPanel


getCSVHeader() - Method in class engine.StatisticsManager


getCSVRow(PottsEngine, Cell) - Method in class engine.CellCoordinatesCSVStatistic


getCSVRow(PottsEngine, Cell) - Method in class engine.CellShapeCSVStatistic


getCSVRow(PottsEngine, Cell) - Method in class engine.CSVStatistic


getCsvWriteFrequency() - Method in class model.Constants


getCurrentCellAreas(Pixel, Pixel) - Static method in class model.AreaEnergyFunction


getCurrentCellPerimeters(Pixel, Pixel) - Static method in class model.PerimeterEnergyFunction


getCurrentPropertiesFilename() - Method in class model.Constants


getCurrentSimulationNumber() - Method in class engine.PottsEngine


getDefaultPixelType(Cell.CellType) - Static method in class model.Pixel


getDeltaEnergy(Pixel, Pixel) - Method in class model.AreaEnergyFunction


getDeltaEnergy(Pixel, Pixel) - Method in interface model.EnergyFunction


getDeltaEnergy(Pixel, Pixel) - Method in class model.EnergyManager


getDeltaEnergy(Pixel, Pixel) - Method in class model.InterfacialTensionEnergyFunction


getDeltaEnergy(Pixel, Pixel) - Method in class model.PerimeterEnergyFunction


getDeltaEnergy(Pixel, Pixel) - Method in class model.PixelInteractionEnergyFunction


getDiagonalNeighborCoordinates(IPositionManager.Position) - Method in class gui.Hexagon.CubeCoordinates


getDisplay() - Method in class model.Cell


getDisplay() - Method in class model.Pixel


getDisplay(int) - Method in class model.Pixel


getDisplayCenterPoint() - Method in class model.Pixel


getDistanceToCell(Cell) - Method in class model.Cell


getEdge(PixelShape.Edge) - Method in class gui.Hexagon


getEdge(PixelShape.Edge) - Method in interface gui.PixelShape


getEdge(PixelShape.Edge) - Method in class gui.Square


getEdgeIndex(PixelShape.Edge) - Method in class gui.HexagonPixelDisplay


getEdgeIndex(int, int, int, int, int) - Static method in class gui.PottsCanvas
:   Returns edge index

getEdgeIndex(PixelShape.Edge) - Method in class gui.SquarePixelDisplay


getEdgeLine(PixelShape.Edge) - Method in class gui.PixelDisplay


getEdgeUnit(PixelShape.Type) - Static method in class model.Pixel


getEnergyFunctions() - Method in class model.EnergyManager


getEnergyType() - Method in class model.AreaEnergyFunction


getEnergyType() - Method in interface model.EnergyFunction


getEnergyType() - Method in class model.InterfacialTensionEnergyFunction


getEnergyType() - Method in class model.PerimeterEnergyFunction


getEnergyType() - Method in class model.PixelInteractionEnergyFunction


getEnergyTypes() - Method in class engine.PottsEngine


getEnergyTypes() - Method in class engine.Utils.EnergyTracker


getEnergyTypes() - Method in class model.EnergyManager


getEngine() - Method in class engine.Statistic


getFilenamePrefix() - Method in class engine.CellCoordinatesCSVStatistic


getFilenamePrefix() - Method in class engine.CellShapeCSVStatistic


getFilenamePrefix() - Method in class engine.CSVStatistic


getFrequency() - Method in class engine.Statistic


getFutureCellAreas(Pixel, Pixel) - Static method in class model.AreaEnergyFunction


getFutureCellPerimeters(Pixel, Pixel) - Static method in class model.PerimeterEnergyFunction


getHSL() - Method in class utils.HSLColor
:   Get the HSL values.

getHue() - Method in class utils.HSLColor
:   Get the Hue value.

getID() - Method in class model.Cell


getInformation() - Method in class model.Cell


getInformation() - Method in class model.Pixel


getInitialLatticeFilename() - Method in class model.Constants


getInteractionEnergy() - Method in class model.Cell


getIsoperimetricQuotient() - Method in class model.Cell


getLastValues() - Method in class engine.Statistic


getLatticeDirectory() - Method in class model.Constants


getLatticeFilename(int) - Method in class model.Constants


getLatticeWriteFrequency() - Method in class model.Constants


getLength() - Method in class model.FiniteVector


getLocalAreaEnergy(Cell, Cell) - Static method in class model.Cell
:   The sum of the area-dependent energies.

getLocalAreaEnergy(Cell, Cell) - Static method in class model.CellSet
:   Gets the area energy of the two cells passed as parameters.

getLocalPerimeterEnergy(Cell, Cell) - Static method in class model.Cell
:   The sum of the perimeter-dependent energies.

getLocalPerimeterEnergy(Cell, Cell) - Static method in class model.CellSet
:   Gets the perimeter energy of the two cells passed as parameters.

getLocalPixelInteractions(Pixel) - Method in interface model.IEnergyFunction
:   Computes the boundary energy around a single pixel.

getLocalTemperature() - Method in class model.Pixel


getLuminance() - Method in class utils.HSLColor
:   Get the Luminance value.

getMaxMcs() - Method in class model.Constants


getMCSCount() - Method in class engine.PottsEngine


getName() - Method in class model.AreaEnergyFunction


getName() - Method in interface model.EnergyFunction


getName() - Method in class model.InterfacialTensionEnergyFunction


getName() - Method in class model.PerimeterEnergyFunction


getName() - Method in class model.PixelInteractionEnergyFunction


getNeighborCells() - Method in class model.Cell


getNeighborCoordinates(PixelShape.Edge) - Method in class gui.Hexagon.AxialCoordinates


getNeighborCoordinates(PixelShape.Edge) - Method in class gui.Hexagon.CubeCoordinates


getNeighborCoordinates(PixelShape.Edge) - Method in class gui.Hexagon


getNeighborCoordinates(PixelShape.Edge) - Method in class gui.Hexagon.OffsetCoordinates


getNeighborCoordinates(PixelShape.Edge) - Method in interface gui.PixelShape


getNeighborCoordinates(PixelShape.Edge) - Method in class gui.Square


getNeighborCoordinates(PixelShape.Edge) - Method in class model.CartesianCoordinates


getNeighborFromPosition(Pixel, IPositionManager.Position) - Method in class model.HexagonPositionManager


getNeighborFromPosition(Pixel, IPositionManager.Position) - Method in interface model.IPositionManager


getNeighborFromPosition(Pixel, IPositionManager.Position) - Method in class model.SquarePositionManager


getNotificationSequenceNumber() - Method in class mvc.AObservable
:   Get the notification sequence number within the source object.

getNotificationSequenceNumber() - Method in interface mvc.IObservable
:   Get the notification sequence number within the source object.

getNotificationSource() - Method in class model.Cell


getNotificationSource() - Method in class model.Lattice


getNotificationSource() - Method in class mvc.AObservable


getNotificationSource() - Method in interface mvc.IObservable


getNumberOfNeighborsFromCell(Cell) - Method in class model.Pixel


getNumberOfNeirestNeighbors() - Method in class model.Constants


getNumberSimulations() - Method in class engine.PottsEngine


getNumEdges() - Method in class gui.HexagonPixelDisplay


getNumEdges() - Method in class gui.PixelDisplay


getNumEdges() - Method in class gui.SquarePixelDisplay


getNumPixelEdges(PixelShape.Type) - Static method in class model.Pixel


getOrigin() - Method in class model.FiniteVector


getOrigin() - Method in class model.HalfLine


getOrigin() - Method in interface model.Vector


getPanel() - Method in class engine.StatisticsManager


getPartialNeighborPositions() - Method in class model.HexagonPositionManager


getPartialNeighborPositions() - Method in interface model.IPositionManager
:   Returns partial neighbors so that iterating over all pixels and getting their respective
    partial neighbors will only result in passing over them a single time.

getPartialNeighborPositions() - Method in class model.SquarePositionManager


getPartialNeighbors(Pixel) - Method in class model.HexagonPositionManager


getPartialNeighbors(Pixel) - Method in interface model.IPositionManager


getPartialNeighbors(Pixel) - Method in class model.SquarePositionManager


getPerimeter() - Method in class model.Cell


getPerimeterEnergy() - Method in class model.Cell
:   Returns perimeter energy such that: energy = perimeterElasticity \* (area
    - targetPerimeter)^2 If any of targetPerimeter or perimeter are negative,
    then returns 0.

getPerimeterEnergy() - Method in class model.CellSet
:   Gets the cumulative perimeter energy of all cells in the system.

getPixel(int, int) - Method in class model.Cell


getPixel(int, int) - Method in class model.Lattice


getPixelColors() - Method in class model.Lattice


getPixelInteraction(Pixel) - Method in class model.Pixel
:   Checks whether pixels are neighbors and then calculates interaction based
    on cell type.

getPixelInteraction(Pixel, Pixel) - Static method in class model.Pixel
:   Checks whether pixels are neighbors and then calculates interaction based
    on cell type.

getPixelIterator() - Method in class engine.PottsEngine


getPixelIterator() - Method in class model.Lattice


getPixelSize() - Method in class model.Constants


getPositionManager() - Method in class model.Lattice


getPositionManager() - Method in class model.Pixel


getProperties() - Method in class model.Constants


getRandomNeighbor(Random) - Method in class model.Pixel


getRGB() - Method in class utils.HSLColor
:   Get the RGB Color object represented by this HDLColor.

getRootDirectory() - Method in class model.Constants


getSaturation() - Method in class utils.HSLColor
:   Get the Saturation value.

getScreenHeight() - Method in class model.Constants


getScreenWidth() - Method in class model.Constants


getSeries(Statistic) - Method in class gui.PlotPanel


getSeriesNames() - Method in class engine.CellEnergyStatistics


getSeriesNames() - Method in class engine.CellStatistic


getSeriesNames() - Method in class engine.CSVStatistic


getSeriesNames() - Method in class engine.DispersionIndex


getSeriesNames() - Method in class engine.EnergyStatistic


getSeriesNames() - Method in class engine.HBLStatistic


getSeriesNames() - Method in class engine.Statistic


getSeriesNames() - Method in class engine.TypeSpecificCellStatistic


getSeriesNames() - Method in class engine.TypeSpecificNearestNeighborStatistic


getSeriesNames() - Method in class engine.TypeSpecificStatistic


getShape() - Method in class gui.PixelDisplay


getShapeIndex(int, int, int) - Static method in class gui.PottsCanvas


getShapeMetric() - Method in class model.Cell


getShapeType() - Method in class gui.Hexagon


getShapeType() - Method in interface gui.PixelShape


getShapeType() - Method in class gui.Square


getSimulationName() - Method in class model.Constants


getSimulationStartTime() - Method in class engine.PottsEngine


getSnapshotDirectory() - Method in class model.Constants


getSnapshotFilename(int) - Method in class model.Constants


getSnapshotWriteFrequency() - Method in class model.Constants


getSpinCopyAttemptCount() - Method in class engine.PottsEngine


getStatsFilename() - Method in class model.Constants


getTarget() - Method in class model.FiniteVector


getTarget() - Method in class model.HalfLine


getTarget() - Method in interface model.Vector


getTimeStampString() - Method in class model.Constants


getTracker() - Method in class model.EnergyManager
:   Makes new tacker based on energy function types.

getType() - Method in class model.Cell


getType() - Method in class model.Pixel


getValidEdges() - Method in class gui.HexagonPixelDisplay


getValidEdges() - Method in class gui.PixelDisplay


getValidEdges() - Method in class gui.SquarePixelDisplay


getVectorTransform(Point) - Method in class model.FiniteVector


getX() - Method in class gui.Hexagon.AxialCoordinates


getX() - Method in class gui.Hexagon.CubeCoordinates


getX() - Method in class gui.Hexagon
:   Get Hexagon center's Cartesian X coordinate.

getX() - Method in class gui.Hexagon.OffsetCoordinates


getX() - Method in class model.CartesianCoordinates


getX() - Method in interface model.Coordinates


getX() - Method in class model.Pixel


getXY(int, int) - Static method in class gui.PottsCanvas


getXYE(int, int) - Static method in class gui.PottsCanvas


getY() - Method in class gui.Hexagon.AxialCoordinates


getY() - Method in class gui.Hexagon.CubeCoordinates


getY() - Method in class gui.Hexagon
:   Get Hexagon center's Cartesian Y coordinate.

getY() - Method in class gui.Hexagon.OffsetCoordinates


getY() - Method in class model.CartesianCoordinates


getY() - Method in interface model.Coordinates


getY() - Method in class model.Pixel


getYAxisLabel() - Method in class engine.CSVStatistic


getYAxisLabel() - Method in class engine.Statistic


getZ() - Method in class gui.Hexagon.AxialCoordinates


getZ() - Method in class gui.Hexagon.CubeCoordinates


getZ() - Method in class gui.Hexagon.OffsetCoordinates


getZ() - Method in class model.CartesianCoordinates


getZ() - Method in interface model.Coordinates


GranerGlazierSimulation - Class in simulations


GranerGlazierSimulation() - Constructor for class simulations.GranerGlazierSimulation


gui - package gui

A B C D E F G H I J K L M N O P R S T U V W

Skip navigation links


- Overview
- Package
- Class
- Use
- Tree
- Deprecated
- Index
- Help

- Prev Letter
- Next Letter

- Frames
- No Frames

- All Classes
